# Value attribution for oncology combination regimens: going beyond frameworks to balance innovation, access, and affordability

**DOI:** 10.3389/fphar.2025.1590944

**Published:** 2025-07-02

**Authors:** Jennifer G. Gaultney, Daniel Ollendorf, Medha Sasane, Céline Fernandez, Krupa Paranjpe, Ting-Yen Chen, Hera Sandhu, Steven Simoens

**Affiliations:** ^1^ IQVIA, London, United Kingdom; ^2^ Center for the Evaluation of Value and Risk in Health, Tufts Medical Center, Boston, MA, United States; ^3^ Sanofi, Bridgewater, NJ, United States; ^4^ Sanofi, Paris, France; ^5^ KU Leuven Department of Pharmaceutical and Pharmacological Sciences, Leuven, Belgium

**Keywords:** value attribution framework, combination therapy access, oncology combinations, value assessment, health technology assessment, reimbursement

## Abstract

Combination therapies are a mainstay in cancer treatment, but reimbursement access can be limited, owing to complexities around value assessment and pricing, and budget impact. Traditional frameworks for value assessment lack specific methodologies for evaluating combinations. A key challenge is value attribution between components. Here we provide the authors’ perspectives on this challenge, along with a summary of current market approaches and two proposed value attribution frameworks (VAFs), including their limitations and what would be needed to apply them in practice. Access to combination therapies varies by country, with each nation adopting different strategies to address challenges. Many have focused their efforts on competition laws, pricing, and overall affordability rather than value attribution. A value attribution solution could provide a basis for pricing and reimbursement negotiations for combinations. The two proposed VAFs offer a possible quantitative solution to assess the value of combination therapy components. However, existing VAFs are still limited by their data requirements and high levels of uncertainty, and are not applicable in certain market archetypes. Further work is needed before such VAFs can be widely applied. In addition, value attribution is only one component of the issue; locally tailored frameworks, agreement on criteria, multi-stakeholder collaboration, and a broader negotiation strategy encompassing other solutions are also necessary. We summarize key challenges and market approaches, as well as factors needed to make the proposed approaches acceptable.

## Introduction

Treating with two or more therapies is becoming increasingly common in oncology, as it helps to address complex factors associated with progression, including drug resistance and tumor heterogeneity (Chen and Song, 2022; Bayat Mokhtari et al., 2017; Valkenburg et al., 2018). Many randomized trials have demonstrated the clinical value of novel combination therapies compared with the current standard of care in oncology, and many more are ongoing (Berger et al., 2023; EFPIA, 2024b). In Europe between 2015 and 2022, approximately 35 combinations were approved for oncologic indications, and many more are expected to launch in the coming years (EFPIA, 2024b); most combination regimens are accessible for patients in Germany, France, and Italy. However, despite demonstrations of the clinical benefits, patient access to approved combinations consisting of two or more novel agents can often be limited or delayed (Latimer et al., 2021a; EFPIA, 2023; EFPIA, 2024b). This limited or delayed access may result from challenges with value assessment or from pricing and reimbursement complexities, particularly in countries like England, Sweden, Finland, Belgium, and the Netherlands, where cost-effectiveness analysis is a key component of reimbursement decisions (EFPIA, 2024b; Dankó et al., 2019; OECD, 2020; OECD, 2024). For many other European countries, such as Cyprus, Latvia, and Malta, access to both single-drug and combination oncology treatments remains low.

Currently, combination treatments are evaluated and priced as single technologies based on the incremental value of the combination as a whole (Briggs et al., 2021). However, this should be done in a way that reflects the respective contributions of components to costs and benefits of combination treatments (OECD, 2020). The total cost of the combination should also reflect overall benefits and be acceptable to payers, as is the case for any treatment. However, existing methods, practices, and policies for assessing value, pricing accordingly, and ensuring a product will be reimbursed are not suitable for combination therapies, which presents systemic challenges for companies, payers, and health technology assessment (HTA) bodies (Dankó et al., 2019; Persson and Norlin, 2018).

One of the key issues in evaluation of novel combination therapies is value attribution, as the clinical value of components may not be purely additive, and currently there is no consensus or defined framework for attributing that clinical value among components of a combination therapy (Latimer et al., 2021a; Kumar et al., 2024). Thus, it is not currently possible to price multiple products within a combination regimen based on their clinical value using a systematic approach. In addition, the backbone therapy is often approved ahead of any add-ons, and the price for that backbone product/component is already set (Latimer et al., 2021b; Kumar et al., 2024). Companies marketing add-on components are responsible for ensuring combinations can be reimbursed. This includes pricing, managing submission to HTA bodies, and ensuring fair attribution of value for the add-on treatment. Market power imbalance between manufacturers and competition law prevents companies from negotiating on price, especially when the manufacturers for the add-on and the backbone products are different (Latimer et al., 2021a; Latimer et al., 2021b). Thus, renegotiating a price that was already agreed is difficult, leading to the combination therapy being deemed not cost-effective, even if priced at zero, as the backbone therapy has a monotherapy-assessed price (equal to the willingness-to-pay [WTP] for its health gain), leaving no room for the add-on therapy to justify its costs as compared to its health gain (Latimer et al., 2021a; Latimer et al., 2021b). Adding to this complexity are issues of prolonged administration of the combination to improve outcomes and multiple indications with potentially different combinations with differing clinical values. Components of a combination regimen are often used across several indications and may be more effective in one cancer type versus another. Similarly, they can be used in multiple ways, such as different treatment lines or stages of disease, or as monotherapy or combined therapy, where the clinical value may also differ. However, it is currently challenging to determine prices that reflect the volume and clinical value of a medicine across different indications or uses. Collectively, these issues may cause pharmaceutical companies to delay seeking reimbursement or create disincentives to invest in add-on treatments, thereby limiting access to innovative treatments at the cost of improving patient outcomes (EFPIA, 2023; Kumar et al., 2024). Stakeholders recognize these challenges, and many efforts have been made to address them, yet access to combination therapies remains limited. One proposed approach is value attribution frameworks (VAFs), which provide a quantitative solution to assess the value of combination therapy components and are primarily relevant within the context of cost-effectiveness-driven HTA frameworks (Kumar et al., 2024; Briggs et al., 2021; Towse et al., 2022). While this would only address the value-attribution part of the problem, such frameworks could inform HTAs and negotiations for some countries (depending on the HTA archetype) and may be an essential tool to support market access of combinations. The proposed VAFs have been in existence for some time now; however, they remain an academic exercise as they are yet to be used in practice by HTAs or payers to support reimbursement decision-making.

We hereby aim to discuss the real-world implications of value attribution approaches for components of a combination therapy and potential unintended consequences of their use, using as examples two published frameworks (Briggs et al., 2021; Towse et al., 2022). We will summarize the authors’ views on value attribution for oncology combinations, covering current challenges and market approaches, and what would be needed to make this approach acceptable.

## Appraisal of two proposed value attribution frameworks

Two distinct quantitative frameworks have been published to date to assess the value attribution of individual components of oncologic combination therapies, utilizing anticipated quality-adjusted life years (QALYs) as a basis for estimation (Briggs et al., 2021; Towse et al., 2022; Steuten et al., 2024). Both frameworks align with decision-making processes of UK’s National Institute for Health and Care Excellence (NICE), based on WTP thresholds for cost-effectiveness. The Briggs framework assesses value attribution of individual components, structured around scenarios where a new add-on is used with an existing backbone therapy. Key market dynamics that are considered in such scenarios include market power, which ascertains whether the manufacturer of one component has more control over pricing than others (imbalanced) or not (balanced), and information availability, i.e., whether the independent benefit of each component is known (perfect information) or not (imperfect information). The model employs a monotherapy ratio approach, which suggests evaluating the combined medications based on QALYs, independent of price, thus addressing the issue of add-on therapies being deemed as not cost-effective at zero price (Briggs et al., 2021; Kumar et al., 2024). The Towse framework, which was recently updated (Steuten et al., 2024), is a more generalized approach compared with that of Briggs. The Towse framework focuses on outcomes and effectiveness of treatments rather than market dynamics (Kumar et al., 2024). In this framework, value attribution is derived as the arithmetic average of the monotherapy and add-on health effect for each product, and the order of backbone and add-on sequence does not impact the value attribution (Towse et al., 2022; Steuten et al., 2024). In cases where the benefits of the add-on or the backbone therapy are unknown, the Towse/Steuten framework recommends using a Bayesian approach to estimate expected outcomes.

While the two value attribution approaches are similar and aim to calculate value shares for constituent therapies using straightforward equations, they differ in underlying assumptions and data requirements, and both approaches result in value attribution with high levels of uncertainty (Table 1) (Gaultney et al., 2023; Kumar et al., 2024). A detailed critical evaluation of both frameworks has been recently published, and it was concluded that, from pharmaceutical manufacturer perspective, the generalized approach of Towse and colleagues was considered most appropriate, as it does not favor any one component of the combination based on the order of market entry. This allows for a slightly more equitable assessment and helps address the challenge of not being cost-effective even at zero price (Kumar et al., 2024). However, the Towse approach still has limitations, such as the requirement for complete information regarding health outcomes, and the need for a Bayesian approach to reflect the uncertainties introduced due to the lack of information. While structured elicitation techniques can be used to solicit missing information (e.g., lack of monotherapy efficacy data for a combination partner), the parameter uncertainties cannot be fully addressed methodologically (Kumar et al., 2024). Such parameter uncertainty is often a challenge in value assessments, while the impact of this uncertainty on value attribution versus the costs of collecting required information is unknown. One of the fundamental issues with value attribution is identifiability, in that, the respective contributions of combination regimens can be determined if their additive effect is linear. In many cases, however, the effect can be sub- or super-additive, so value attribution calculations are less clear due to the uncertain contribution of each component.

**TABLE 1:** Limitations of the briggs and towse frameworks.

Aspect	Approach by Briggs framework	Approach by Towse framework
Independence of monotherapy benefit from other components	The Briggs framework is feasible when the monotherapy value of the add-on is unknown. In such cases, the value attribution of the add-on will be dependent on both the backbone’s benefit as a monotherapy and the combination therapy’s benefit	The Towse framework requires monotherapy data of all components. Value attribution is based on the individual monotherapy benefit and the combination benefit. Towse proposes the use of a Bayesian approach to estimate the expected outcomes of the component for which there is no data
Market power assumption	Briggs assumes an imbalanced market when the manufacturer of one drug may have more control, or “market power”, over pricing decisions than the manufacturer of other drugs. The Briggs value attribution framework reflects “market power” by means of a weighting parameter	In the Towse framework, the remaining value of the combination therapy, after accounting for the monotherapy value, is divided equally among the constituents rather than according to the market power. However, the Towse framework can be flexible to give more weight to certain constituents rather than evenly allocating weight
Satisfaction of universal and symmetrical principles	Under the perfect information scenario, the Briggs framework is not universal; it does not consider each constituent’s contribution to the combination regimen. Under the imperfect information scenario, the Briggs framework is not symmetrical; it can lead to undervaluing the add-on in sub-additive or overvaluing it in super-additive scenarios	The Towse framework solves for value attribution between the outcomes derived from the perfect and imperfect scenarios in the Briggs framework. It weighs both the relative contributions of the products as monotherapies and what they add as an incremental therapy to a combination

## Current market approaches to providing access to combination therapies

Across Europe to date, a variety of approaches have been suggested to tackle pricing and reimbursement issues for combination therapies (OECD, 2020), yet none fully address market access challenges for such products (Table 2). Value assessment approaches seem to focus on efforts to address competition laws, pricing, and overall affordability rather than the issue of value attribution.

**TABLE 2:** Country-specific approaches for combination therapies^a^.

Country	Current situation
Cost-effectiveness driven	
Sweden	• The Swedish Institute for Health Economics has proposed IBP as a potential solution, suggesting that the pricing of combinations should reflect their value in each specific indication ((Lif), 2023) • The TLV is developing a pricing strategy for combinations, incorporating input from relevant stakeholders, such as the industry and regions (OECD, 2020) • The New Therapies Council proposed a few initiatives to facilitate access to combination therapies, including a voluntary commitment for interested groups, mandatory sharing of certain health economic data, and the application of different prices for different uses during health economic evaluation ((Lif), 2023, EFPIA, 2024a)
UK	• NICE does not attribute value between combination therapy components; this must be done prior to submission (OECD, 2020) • ABPI has a dedicated working group to address the issues associated with combination therapies (ABPI, 2023) • Following engagement with the CMA, NICE, and NHSE, the ABPI proposed a cross-company pricing negotiation framework at the end of 2023 to ensure cost-effective access to combination treatments while complying with competition laws (ABPI, 2023; CMA, 2023; EFPIA, 2024a; OECD, 2020) • At the end of 2023, the CMA made a ‘first of its kind’ statement detailing the circumstances under which it would not investigate the exchange of information or commercial agreements between competing pharmaceutical companies with regards to providing combination therapies for NHS patients (CMA, 2023; EFPIA, 2024a) • More work is needed to address remaining barriers, including lack of an HTA framework for combinations and implementation of flexible pricing methods for components within combinations (ABPI, 2023) • ABPI is exploring the feasibility of two proposed VAFs (ABPI, 2023)
Ireland	• There is no established formal mechanism specific for combination reimbursement, and the process is based on negotiations between payers and sponsors involved, similar to other technologies • Throughout 2023, IPHA was working to establish a legal framework which facilitates member companies to compliantly negotiate prices of combinations using frameworks approved under local competition law (EFPIA, 2024a)
Budget-impact driven	
Spain	• Reimbursement is only officially granted after a satisfactory price has been negotiated; manufacturers may have to provide significant discounts (Izmirlieva, 2021) •FarmaIndustria stressed the importance of establishing specific evaluation and pricing frameworks for combination treatments and proposed implementing the IBP mechanism (FarmaIndustria, 2023)
Italy	• Several types of MEAs may be applied to the product used in the combination by implementing a patient monitoring registry (OECD, 2020) • When one medication in the combination is already covered by the Italian NHS, its price is considered a fixed parameter, and the price of the second component is determined to reflect the added value of the combination (OECD, 2020)
Belgium	• Multi-indication pricing models are being discussed, which would allow price adjustment according to volume and value of the medicine in real-life practice across indications, although these are not combination therapy-specific (Maes et al., 2023) • Toward the end of 2023, the Belgian pharmaceutical trade association published a newsletter calling on all stakeholders, including the Belgian Competition Authority, to get involved in finding a solution (PharmaBe, 2023) • A “mirroring reimbursement procedure” has been proposed, to ensure the involvement of all combination therapy manufacturers during the pricing and reimbursement process, and to ensure simultaneous reimbursement and patient access timeline for all components in a combination treatment (EFPIA, 2024a; PharmaBe, 2023). oThe proposed procedure provides backbone companies with more involvement and negotiation possibilities and avoids solely placing the burden of incremental budget on either the add-on or backbone therapy. • INAMI is proposed as the central decision-maker (PharmaBe, 2023) • A working party has been established between the healthcare payer, the pharmaceutical industry, and the Belgian Competition Authority to tackle the issue of market access of combination therapies (Riziv-inami, 2023)
Clinical-benefit driven	
France	• If the combination has a minor added benefit, cost cannot exceed that of the comparator; if there is moderate or major added benefit, the combination price may be set at 10% or higher (OECD, 2020) • France conducts one-on-one negotiations with companies, considering a pre-determined acceptable price for combinations, to address competition law barriers (OECD, 2020) • A share of the ‘value’ is not attributed to the constituents (OECD, 2020)
Germany	• The GKV Financial Stabilization Act was passed in November 2022, introducing a special rebate of 20% on the total costs of combination therapies launched since 2011 with data protection (in effect from May 2023) (Engelke et al., 2023) • Significant issues reported early in 2024 by the first implementation of the 20% rebate rule (Melke, 2024) • In October 2024, the BMG issued guidance on which medicines/combinations would qualify for the rebate, but not all stakeholders appear to be in agreement (Für gesundheit, 2024; Navlin, 2024)

^a^ Table based on publicly available information at the time of submission.

ABPI, association of the british pharmaceutical industry; BMG, Bundesministerium für Gesundheit (Federal Ministry of Health [Germany]); CMA, Competition & Markets Authority; GKV, gesetzliche Krankenversicherung (statutory health insurance [Germany]); IBP, indication-based pricing; INAMI, Institut national d’assurance maladie-invalidité (National Institute for Health and Disability Insurance [Belgium]); IPHA, irish pharmaceutical healthcare association; MEA, managed entry agreements; NICE, national institute for health and care excellence; NHSE, national health service england; TLV, Tandvårds-och läkemedelsförmånsverket (Dental and Pharmaceutical Benefits Agency [Sweden]).

The variety of approaches across countries may be due to inherent differences in healthcare systems, policies, and market archetypes for assessing the value, pricing, and reimbursement of new medicinal products. In markets that use the cost-effectiveness archetype, such as Sweden, UK, and Ireland, medicines are evaluated by comparing costs to delivered health outcomes. Health outcomes are measured using well validated matrix metrics, such as QALYs or quantifiable clinical outcomes (e.g., number of heart attacks prevented) (Frisell and Steen Carlsson, 2024; Persson and Norlin, 2018). A key challenge in these markets is balancing perceived value and cost within the cost-effectiveness framework according to the healthcare system’s WTP threshold. For combination treatments associated with an increased treatment duration, if the backbone treatment cost (inclusive of confidential discounts) is at or near the payer’s WTP and cannot be renegotiated, the cost of the add-on treatment is unlikely be covered within the WTP threshold (Latimer et al., 2021a). This may render the combination therapy as not cost-effective, even if the add-on therapy has a price of zero. Countries using this approach therefore can face challenges in assessing double-branded drug combinations as cost-effective, resulting in lack of reimbursement and of access to novel therapies that have the potential to improve outcomes. On the other hand, when the combination treatment is associated with the same or even a reduced duration, its added value is not consumed by the incremental cost of the backbone therapy and can be attributed solely to the combination partner.

Recently in the UK, a negotiation framework has been proposed which permits a dialog between two competing companies to exchange information and enter commercial agreements on combination therapies (CMA, 2023; EFPIA, 2024a). However, how this would be facilitated and by whom remains unclear. The UK is also exploring the feasibility of incorporating the Briggs or Towse VAFs (ABPI, 2023) in NICE assessments to further expand methodological approaches to evaluate combination treatments. Other countries using a cost-effectiveness framework for reimbursement are also embarking on similar initiatives. For example, in Sweden, a 2023 joint report between the New Therapies Council and the Swedish Pharmaceutical Industry Association (Läkemedelsindustriföreningen [Lif]) proposed a new model that relies on voluntary collaboration between interested stakeholders to promote access to combinations (Lif, 2023; EFPIA, 2024a). A pilot project was also proposed to identify suitable candidate combinations for consideration through horizon scanning, which identifies new treatments in development, but to our knowledge, this has either not been implemented or is yet to be published.

Countries that use a budget-impact approach, such as Spain and Italy, also face similar challenges, even though the approach to value assessment and subsequent reimbursement is different. In these countries, the focus is on the financial impact a new medicine would have on the healthcare budget and the financial implications for payers (Izmirlieva, 2021). As with the cost-effectiveness approach, often there is minimal room for additional costs, and renegotiation to accommodate the cost of the add-on product remains challenging. However, this assumes that what is considered affordable is transparent and quantifiable, which is not always the case for markets driven by cost-effectiveness and budget impact. For example, while Belgium applies a cost-effectiveness criterion to HTA decision-making, no WTP threshold is prespecified, and oncology combinations are mainly assessed from a budget-impact and clinical-benefit perspective.

While countries such as France and Germany assess value of treatments separately compared with the available current standard of care in those countries, value assessment of combination treatments can be challenging due to the lack of mature or comparative data versus standard of care. The clinical-benefit approach assesses efficacy, safety, and improvements in quality of life, and considers whether there is a significant clinical advantage over existing treatments. For therapies with uncertain benefits irrespective of a combination partner, this challenge is at times being overcome in Germany and France, and arguably many other countries such as the UK, through approaches like managed entry agreements and coverage with evidence development. In France, once the WTP threshold has been determined, negotiations with the individual companies by the pricing committee take place to adjust the price (OECD, 2020). Value attribution is not calculated and has not been the focus of efforts to improve overall access but could potentially lead to more efficient and equitable pricing negotiations.

The efforts being made across countries to improve methods for pricing and reimbursement of combination therapies are encouraging. However, the absence of a legal framework is broadly accepted as a main limiting factor (Latimer et al., 2021a). Much more needs to be done to facilitate cross-company discussions without the risk of breaking competition laws.

Considering the differences across countries, it is clear that no one solution for improving access to combination therapies in oncology will be suitable for all, particularly with regards to value attribution. Countries are in different phases of adoption, but even those who are most advanced are far from having an effective, practical approach to value attribution.

## Improving value attribution for oncology combination therapies: a time for change

As of August 2022, there were 77 Phase 2 and 3 trials planned for oncology combination therapies (EFPIA, 2024a); thus, the need to have a framework in place to assess these combinations, if and when approved, is urgent. While two elegant frameworks have been proposed, they are underused. A key issue may be that the importance of value attribution in the process of reimbursing combination therapies in oncology still does not appear to be fully recognized. In addition, no specific guidelines or legal frameworks support use of VAFs, which still seem to be considered exploratory.

The VAFs that have been proposed so far may over-index the backbone treatment, undervaluing the contribution of the add-on, a limitation that is seen with the Briggs framework. With no incentives for add-on therapies and no mechanism for incorporating incremental benefit, existing VAFs may not fully recognize the value of an innovative treatment. In addition, the impact of generic entry, which would affect the overall cost-effectiveness of a combination therapy and could have implications for pricing of both components, is not considered in these VAFs. Value attribution is also sensitive to monotherapy outcomes. The proposed quantitative approaches require information on monotherapy benefits or estimates of market power; if these data are not available or are uncertain, the framework outcomes would be inaccurate. However, many add-ons are not studied or marketed as monotherapies, as this was never their intended use. New components of multi-drug regimens are often first studied in single-arm trials, so the ability to assess their comparative benefit (versus standard of care) is limited.

Despite limitations, VAFs are a step forward in understanding and quantifying value (and price) of combination therapy components with a focus on a common value indicator (WTP/QALY). However, such solutions are only indicative and not conclusive from a price-negotiation perspective due to inherent uncertainties discussed earlier. Qualitative solutions could facilitate management of clinical and economic benefit uncertainty (e.g., using real-world research and improving clinical development programs), but this requires a wider acceptance of real-world evidence in medicine approvals, pricing, and reimbursement processes (Vancoppenolle et al., 2023).

In addition, locally tailored frameworks, agreement on value assessment criteria, and broader negotiation strategies encompassing other solutions are necessary. The currently available VAFs are more relevant in markets driven by cost-effectiveness and less so for those driven by budget impact or clinical benefit, so a multidimensional approach is needed. Local VAF adaptation may represent a more suitable approach to increase patient access to therapy by accounting for differences across markets, but this will require payers and industry and methodology experts to develop local solutions. Indeed, VAFs are a multi-stakeholder matter, and guidance could be produced under the umbrella of an independent organization (e.g., ISPOR).

To ensure the discussed changes can be implemented, sound legal frameworks are also required. These would need to be relevant for each market, to account for any differences in local laws, policies, and methods.

## Future perspectives

The path to treating cancer increasingly involves use of novel combination therapies to address complex factors, including drug resistance and tumor heterogeneity, that evade monotherapy approaches. The development of VAFs for reimbursement of such therapies represents a significant advancement in efforts to improve patients’ access to life-saving combination therapies. Value attribution may help to inform pricing of components of a combination therapy. However, given the current state of existing VAFs and their limitations, the outcome of such frameworks is indicative rather than conclusive, because they do not provide an entirely solid, robust, or valid value split. The importance of the value attribution aspect of the access issue needs to be fully recognized, and VAFs will require further refinement and validation, but wider efforts are also required to resolve the challenge of limited access to combination therapies in oncology. VAFs are only one of many factors that need to be taken into consideration; a holistic approach considering all factors is needed (e.g., indication-based pricing, competition law arbitration). Policies need to be revised to focus on providing access, and case-by-case solutions should be available based on unmet needs.

The landscape for market access of oncology combination therapies is evolving, with different initiatives being undertaken at national levels. Despite this evolution, patient access to novel combination treatment regimens remains a key challenge. A collaborative approach across all stakeholders is required to overcome systemic issues—health system administrators should be involved in payer value attribution discussions, along with healthcare providers and health economists. Efforts should also involve sponsors of combination treatments, patients, trade unions, policymakers, and, possibly, regulatory bodies. Overarching bodies/associations can provide a neutral platform to aggregate positions from different stakeholders. A long-term commitment at a government level to secure access to combination regimens is also important to allow time for policy level changes. Joint systemic solutions may require new regulations, as currently there are no comprehensive, specific guidelines or legal frameworks supporting evaluation of combination therapies. However, these will need to be adapted for different countries. Already, a lack of unified guidelines means that use of combination therapies across countries is not equal (EFPIA, 2024a; Vancoppenolle et al., 2023). The path to enhancing cancer outcomes lies in the use of combination therapies, and attribution of value is crucial for ultimately ensuring patient access.

## Data Availability

The original contributions presented in the study are included in the article/supplementary material, further inquiries can be directed to the corresponding author.
